# Recovery of *Bacteroides thetaiotaomicron* ameliorates hepatic steatosis in experimental alcohol-related liver disease

**DOI:** 10.1080/19490976.2022.2089006

**Published:** 2022-07-03

**Authors:** Moris Sangineto, Christoph Grander, Felix Grabherr, Lisa Mayr, Barbara Enrich, Julian Schwärzler, Marcello Dallio, Vidyasagar Naik Bukke, Archana Moola, Antonio Moschetta, Timon E. Adolph, Carlo Sabbà, Gaetano Serviddio, Herbert Tilg

**Affiliations:** a Department of Internal Medicine I, Gastroenterology, Hepatology, Endocrinology & Metabolism, Medical University Innsbruck, Innsbruck, Austria; b C.U.R.E. (University Center for Liver Disease Research and Treatment), Liver Unit, Department of Medical and Surgical Sciences, University of Foggia, Foggia, Italy; c Department of Precision Medicine, University of Campania “Luigi Vanvitelli”, Naples, Italy; d Department of Interdisciplinary Medicine, University of Bari, Bari, Italy

**Keywords:** Alcohol-related liver disease, Bacteroides thetaiotaomicron, microbiota, steatosis, intestinal barrier, mitochondria

## Abstract

Alcohol-related liver disease (ALD) is a major cause of liver disease and represents a global burden, as treatment options are scarce. Whereas 90% of ethanol abusers develop alcoholic fatty liver disease (AFLD), only a minority evolves to steatohepatitis and cirrhosis. Alcohol increases lipogenesis and suppresses lipid-oxidation implying steatosis, although the key role of intestinal barrier integrity and microbiota in ALD has recently emerged. *Bacteroides thetaiotaomicron* (*Bt*) is a prominent member of human and murine intestinal microbiota, and plays important functions in metabolism, gut immunity, and mucosal barrier. We aimed to investigate the role of *Bt* in the genesis of ethanol-induced liver steatosis. *Bt* DNA was measured in feces of wild-type mice receiving a Lieber-DeCarli diet supplemented with an increase in alcohol concentration. In a second step, ethanol-fed mice were orally treated with living *Bt*, followed by analysis of intestinal homeostasis and histological and biochemical alterations in the liver. Alcohol feeding reduced *Bt* abundance, which was preserved by *Bt* oral supplementation. *Bt*-treated mice displayed lower hepatic steatosis and triglyceride content. *Bt* restored mucosal barrier and reduced LPS translocation by enhancing mucus thickness and production of Mucin2. Furthermore, *Bt* up-regulated *Glucagon-like peptide-1* (GLP-1) expression and restored ethanol-induced *Fibroblast growth factor 15* (FGF15) down-regulation. Lipid metabolism was consequently affected as *Bt* administration reduced fatty acid synthesis (FA) and improved FA oxidation and lipid exportation. Moreover, treatment with *Bt* preserved the mitochondrial fitness and redox state in alcohol-fed mice. In conclusion, recovery of ethanol-induced *Bt* depletion by oral supplementation was associated with restored intestinal homeostasis and ameliorated experimental ALD. *Bt* could serve as a novel probiotic to treat ALD in the future.

## Introduction

The excessive alcohol introit represents a social, political, and health-care issue worldwide. In Europe and North America, alcohol-related liver disease (ALD) is the main cause of liver disease and severe alcoholic hepatitis (AH), implying high morbidity and mortality rates.^1–3^ Chronic alcohol consumption provokes the development of hepatic steatosis in 90% of alcoholics, while 10–35% of these evolve to alcoholic steatohepatitis (ASH) and finally only 10–20% develop cirrhosis.^4,5^ Therefore, ethanol drinking can be seen as the first step in the genesis of ALD, which is still reversible. The enzymatic metabolism of ethanol diminishes the NAD^+^/NADH ratio with consequent enhancement of fatty acid (FA) synthesis and suppression of FA oxidation (FAO).^6,7^ Accordingly, studies demonstrated that alcohol assumption enhances the expression of genes involved in lipogenesis, such as *Fatty acid synthase* (FASN) and *Stearoyl-CoA desaturase-1* (SCD-1), while β-oxidation is dampened likely due to inhibition of *Peroxisome proliferator-activated receptor-α* (PPAR-α) activity.^8–12^ However, the pathogenesis of ALD is very complex, and recently the key role of intestinal homeostasis has emerged. In fact, ethanol ingestion provokes microbiota perturbations and disruption of barrier function, which lead to translocation of pathogen-associated molecular pattern molecules (PAMPs) (e.g. lipopolysaccharide, LPS) and activation of Kupffer cells with consequent inflammation and oxidative stress in the liver.^13,14^ Moreover, some studies have showed that intervention in microbiota seems to protect against ethanol-induced liver injury.^15–17^ *Bacteroides thetaiotaomicron* (*Bt*) is a gram-negative anaerobe and a prominent member of intestinal microbiota in humans and mice. *Bt* constitutes 6% of all intestinal bacteria and 12% of all *Bacteroides* in humans, and exerts important functions in mucosal barrier, immunity, and nutrient metabolism.^18,19^ It has been reported that the commensal *Bt* crucially drives the mucosal barrier development during weaning and also protects from hyperpermeability in an inflammatory contest.^20,21^ Since an impaired intestinal barrier reflects an important pathogenetic player in ALD, we have hypothesized a loss of this commensal after alcohol assumption. Moreover, a recent study associated *Bt* depletion with obesity, while *Bt* administration protected high-fat fed mice against obesity by promoting lipolysis in adipose tissue.^22^ Therefore, considering the potential role of *Bt* in modulation of lipid metabolism and its importance in barrier function maintenance, we investigated whether *Bt* intestinal abundance is impaired by alcohol assumption and whether its supplementation implies metabolic beneficial effects in the liver.

## Results

### *Ethanol reduced* Bt *amount in vivo and in vitro*

**Figure 1.**
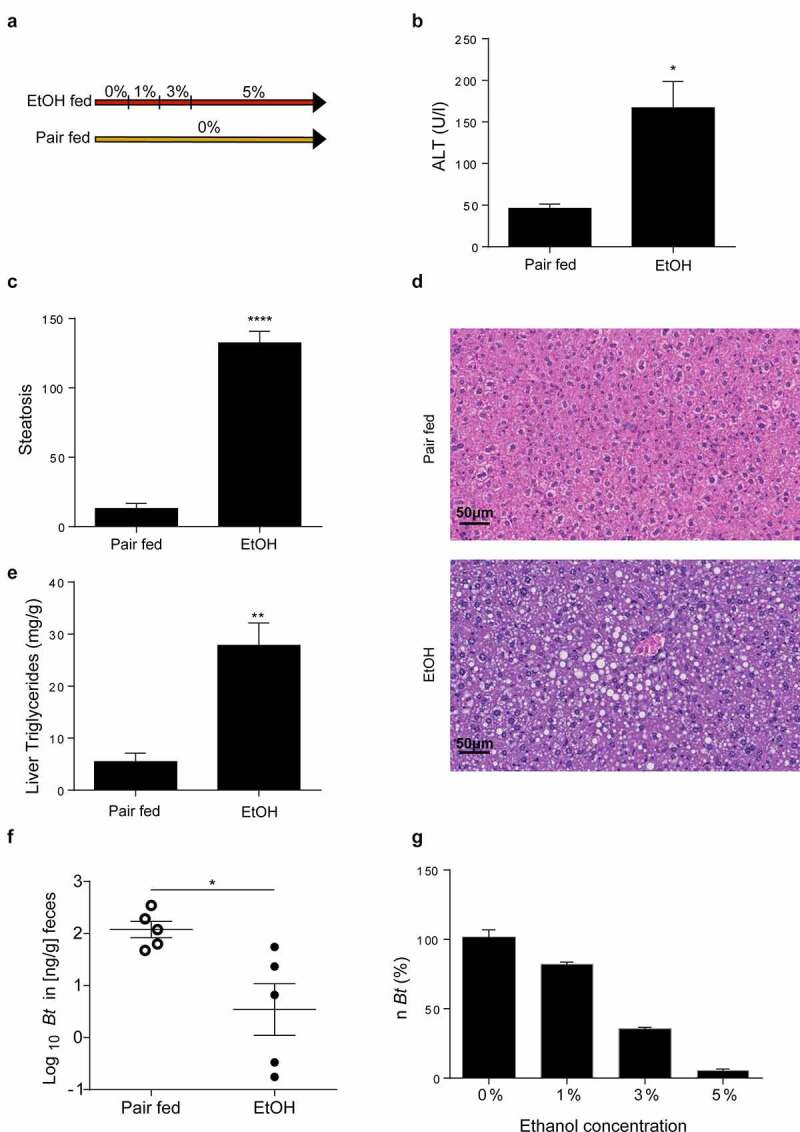
Ethanol depletes *Bt*. (a) Design of Lieber-DeCarli model. (b) Serum ALT levels (Pair fed = n6; EtOH = n10). (c, d) Histological determination of hepatic steatosis with representative pictures of H&E staining (n = 5 per group). (e) Liver triglyceride content (Pair fed = n5; EtOH = n6). (f) Quantification of *Bt* DNA in feces measured by qPCR (n = 5 per group). (g) Quantification of *Bt* number (expressed in percentage) in LYBHI medium supplemented with different ethanol concentrations. Data are expressed in mean ± SEM; *p < .05, **p < .01, ***p < .001, ****p < .0001 according to two-tails student’s t-test.

Abbreviations: *Bt, Bacteroides thetaiotaomicron*.

In order to investigate whether ethanol impacts the *Bt* abundance in the intestine, mice were fed an ethanol-containing diet (Lieber-DeCarli diet, EtOH-fed) for 15 days (Figure 1a). Ethanol feeding resulted in increased ALT levels and liver triglyceride accumulation with steatosis development, hallmarks of ALD (Figure 1 b-e).

Importantly, we note that ethanol feeding induced a significant reduction in *Bt* compared to pair-fed mice (figure 1f). This was in line with *in-vitro* study, where *Bt* growth was inhibited by increasing ethanol concentrations (Figure 1g).

### Bt *supplementation ameliorated hepatic steatosis*

**Figure 2.**
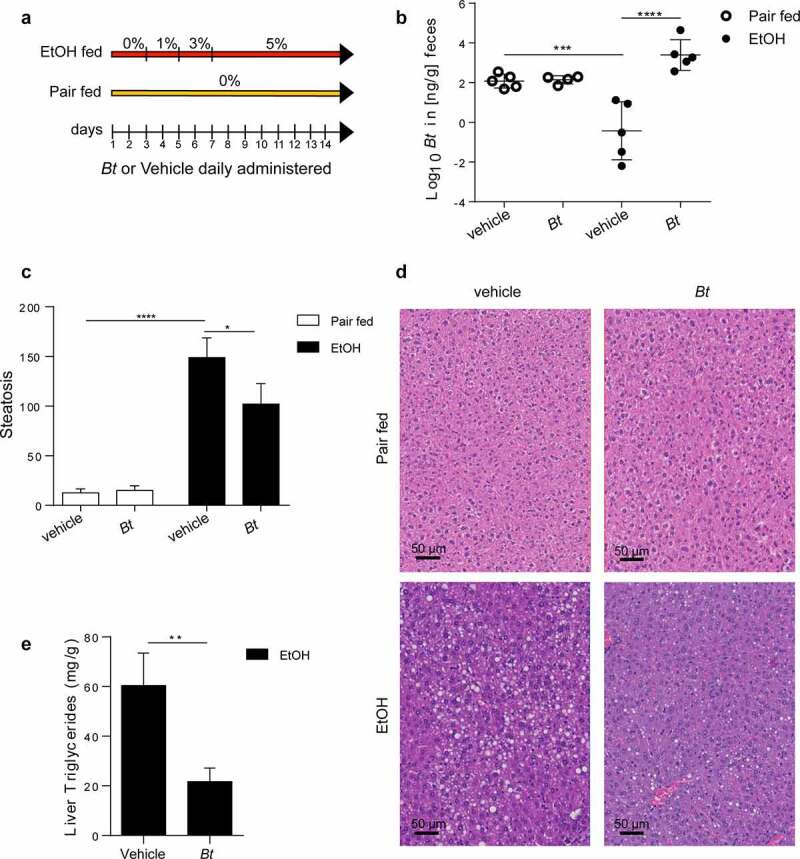
*Bt* supplementation ameliorates alcoholic fatty liver disease. (a) Experimental design. (b) Quantification of *Bt* DNA in feces measured by qPCR (Pair fed groups = n4-5; EtOH groups = n5). (c, d) Histological determination of hepatic steatosis with representative pictures of H&E staining (Pair fed groups = n6; EtOH groups = n9). (e) Liver Triglyceride content in EtOH fed mice (n = 9–10). Data are expressed in mean ± SEM; *p < .05; **p < .01; ***p < .001, ****p < .0001 according to one-Way ANOVA followed by post hoc analysis (Bonferroni test) or two-tails student’s t-test.

Abbreviations: Bt, Bacteroides thetaiotaomicron.

In the next step, ethanol-fed mice were treated with *Bt* by oral gavage (Figure 2a). Bacterial supplementation was effective to recolonize *Bt* in EtOH-fed mice (Figure 2b). Notably, although the two ethanol fed groups showed the same alcohol assumption (Supplementary Fig.1), *Bt*-treated mice presented a grade of hepatic steatosis significantly lower (Figure 2c, d) compared to vehicle treated mice. Moreover, this observation was corroborated by the significant reduction of triglycerides (TG) content in the liver of EtOH-fed *Bt*-treated mice (Figure 2e). Meanwhile, no difference was found in serum ALT levels, and *Bt* only mildly reduced IL1β expression and neutrophilic infiltration in the liver (Supplementary Fig.2).

### Bt *preserved mucosal barrier function*

**Figure 3.**
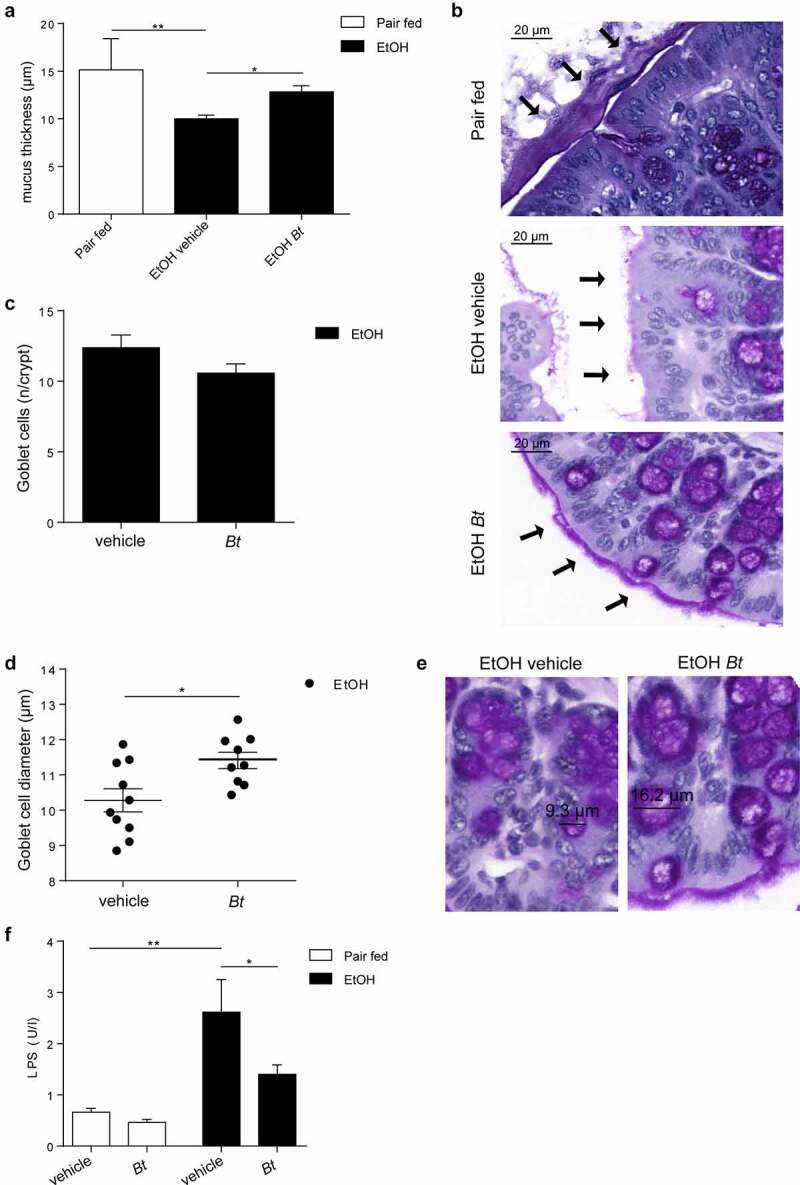
*Bt* recovery restores mucus production in the colon. (a, b) Quantification of mucus thickness in colon sections stained with periodic acid–Schiff reaction (Pair fed = n4; EtOH groups = n9-10). (c) Number of goblet cells per crypt and (d, e) goblet cells diameter identified by periodic acid–Schiff reaction. (f) Serum LPS concentration (Pair fed groups = n6; EtOH groups = n6-8). Data are expressed in mean ± SEM; *p < .05; **p < .01; ***p < .001, ****p < .0001 according to one-Way ANOVA followed by post hoc analysis (Bonferroni test) or two-tails student’s t-test.

Abbreviations: *Bt, Bacteroides thetaiotaomicron*, LPS, lipopolysaccharide.

**Figure 4.**
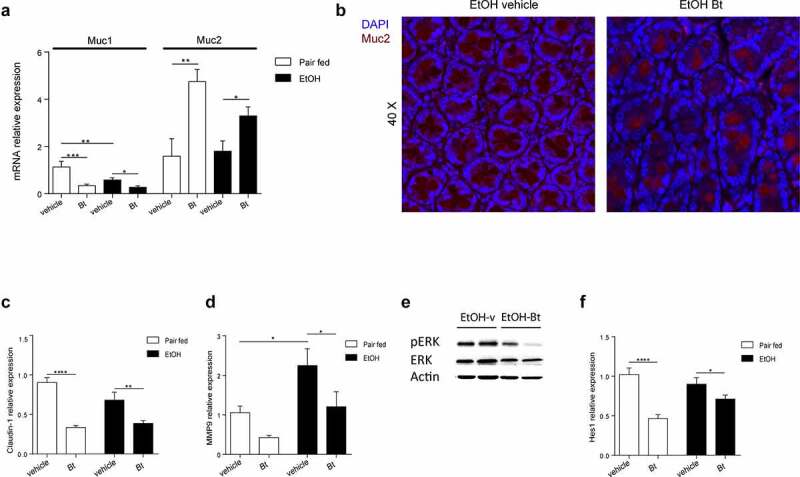
*Bt* promotes mucin2 production via downregulation of claudin1-Notch axis (a) Colonic expression of muc1 and muc2 fold over *Pair fed vehicle group* and determined by qPCR (Pair fed groups = n6; EtOH groups = n10). (b) Muc2 immunoreactivity (red) with representative confocal microscope image of murine colon in EtOH-fed mice. DAPI, blue. (c-d) Colonic expression of Claudin1 and MMP9 fold over *Pair fed vehicle group* and determined by qPCR (Pair fed groups = n5-6; EtOH groups = n9-10). (e) Representative pictures of protein levels of actin, ERK and pERK determined by western blot analysis (EtOH groups = n5). (f) Colonic expression of Hes1 fold over *Pair fed vehicle group* and determined by qPCR (Pair fed groups = n5-6; EtOH groups = n9-10). Data are expressed in mean ± SEM; *p < .05; **p < .01; ***p < .001, ****p < .0001 according to one-Way ANOVA followed by post hoc analysis (Bonferroni test).

Abbreviations: Muc1, mucin1; muc2, mucin2; DAPI, 4′,6-diamidino-2-phenylindole; MMP9, matrix metallopeptidase 9; ERK, extracellular signal-regulated kinase 1/2; pERK, phosphorylated ERK. Hes1, hairy and enhancer of split-1; *Bt, Bacteroides thetaiotaomicron*.

Treatment with *Bt* partially restored the ethanol-induced mucus disruption as shown by higher mucus thickness in PAS-stained colon sections (Figure 3a, b). Although, *Bt* administration was not associated with altered number of goblet cells in EtOH-fed mice, we could observe a significant increase in goblet cell diameter (Figure 3c-e). It is important to note that lower levels of circulating LPS were detectable in EtOH-fed *Bt*-treated mice (figure 3f) compared to EtOH-vehicle, suggesting a restored gut barrier function. To further elucidate the role of *Bt* in mucosal preservation in the colon, we found that Mucin1 (Muc1) was transcriptionally downregulated, while Mucin2 (Muc2) expression was enhanced by *Bt*, corroborated by Muc2 immunofluorescence (Figure 4a, b). Muc2 is the main component of mucus layer and probably *Bt* promoted mucus production by increasing muc2. Therefore, ethanol decreased the total mucus amount compared to *Bt*, which restored the layer of mucus by acting mostly on muc2 production. Although mRNA levels do not show different muc2 expression between pair fed and ethanol, the muc2 histological staining clearly shows the lack of mucin2 in the ethanol vehicle mice. However, we recognize that several other types of mucins could be involved. The integrity of epithelial tight junctions is also important in preserving the barrier function, thus its alteration is crucial in ethanol-related leaky gut.^14,23,24^ Therefore, we evaluated the expression profile of tight junction proteins, finding a significant down regulation of Claudin 1 (Cldn1) transcription in *Bt-*treated mice (Figure 4c). Cldn1 over-expression in colon inflammation has been associated with higher MMP9 transcription and ERK phosphorylation to induce Notch activity with the consequent muc2 suppression.^25^ Accordingly, we found that *Bt* administration reduced MMP9 expression, ERK phosphorylation, and Hes1 expression (transcriptional marker of Notch activity) (Figure 4d-f). In addition, MMP9 was upregulated by ethanol (Figure 4d).

The increased expression of Reg3γ and Reg3β lectins also underlined an immunological effect of *Bt* administration in EtOH fed mice (Supplementary Fig 3).

### Bt *modulated GLP1 and FGF15 expression*

**Figure 5.**
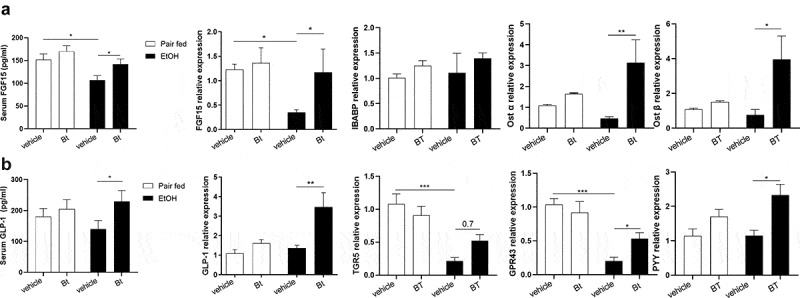
*Bt* modulated GLP1 and FGF15 expression. (a) Serum levels of FGF15 and ileal expression of FGF15, IBABP, Ost-α and Ost-β fold over *Pair fed vehicle group* and determined by qPCR (Pair fed groups = n4-6; EtOH groups = n6-10). (b) Serum levels of GLP-1 and colonic expression of GLP-1, TGR5, GPR43 and PYY fold over *Pair fed vehicle group* and determined by qPCR (Pair fed groups = n4-6; EtOH groups = n6-10). Data are expressed in mean ± SEM; *p < .05; **p < .01; ***p < .001, according to one-Way ANOVA followed by post hoc analysis (Bonferroni test).

Abbreviations: FGF15, fibroblast growth factor 15; IBABP, ileal bile acid-binding protein; Ost-α/β, Organic solute transporter α/β; GLP-1, Glucagon-like peptide-1; G protein-coupled bile acid receptor 1; GPR43, G-protein-coupled receptor 43; Peptide YY; *Bt, Bacteroides thetaiotaomicron*.

Intriguingly, *Bt* modulated intestinal hormone production as *Bt* enhanced GLP-1 expression and restored the ethanol-induced FGF15 downregulation (Figure 5a, b), in the colon and ileum, respectively. The serum levels of GLP-1 and FGF-15 corroborated these findings (Figure 5a, b). Supposing the role of the intestinal FXR in FGF15 dysregulation, the expression of other FXR targets was measured (i.e., IBABP, Ost-α, and Ost-β), underlying a decreased expression in EtOH-vehicle mice and an increased expression (significant for Ost-α and Ost-β) in EtOH-*Bt* mice (Figure 5a). These findings highlight the intestinal FXR dysregulation in ALD, while the administration of *Bt* improved FXR function probably by modulating bile acid metabolism or by the production of unknown FXR activators. Accordingly, in the liver, the expression of CYP7A1, a major bile acid synthesis modulator typically downregulated by FXR and FGF15-FGFR4, was significantly up-regulated in EtOH-fed mice and normalized in *Bt*-treated mice (supplementary Fig.4).

When analyzing the GLP-1 induction pathways in the colon, we found that GPR43, a SCFA receptor, and TGR5, a bile acid receptor, were both downregulated in EtOH-fed mice and restored by *Bt* treatment (Figure 5b). GPR43 and TGR5 induce prompt GLP-1 and PYY production;^26^ accordingly, also PYY was up-regulated in EtOH-*Bt* mice (Figure 5b). The modulation of both receptors underlines that bile acid homeostasis together with bacterial SCFA could contribute to GLP-1 production in *Bt*-treated mice.

### Bt *improved hepatic metabolism*

**Figure 6.**
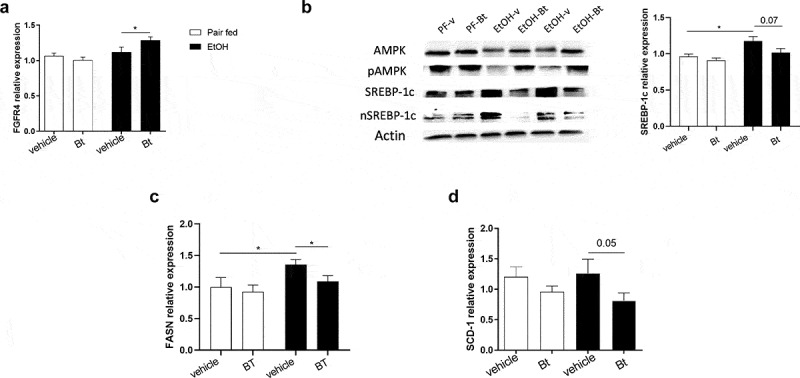
*Bt* reduced hepatic FA synthesis (a) Liver expression of FGFR4 fold over *Pair fed vehicle group* and determined by qPCR (Pair fed groups = n5-6; EtOH groups = n9-10). (b) Representative pictures of protein levels of actin, AMPK, pAMPK, SREBP-1c, and nuclear SREBP-1c, determined by western blot analysis and expression of SREBP-1c determined by qPCRin the liver (for western blot: Pair fed groups = n3; EtOH groups = n5; for qPCR: Pair fed groups = n5-6; EtOH groups = n9-10). (c, d) Liver expression of FASN and SCD-1 fold over *Pair fed vehicle group* and determined by qPCR (Pair fed groups = n4; EtOH groups = n8-10). Data are expressed in mean ± SEM; *p < .05, according to one-Way ANOVA followed by post hoc analysis (Bonferroni test).

Abbreviations: FA, fatty acids; FGFR4, Fibroblast Growth Factor Receptor 4; AMPK, 5’ AMP-activated protein kinase; pAMPK, phosphorylated AMPK; SREBP-1c, Sterol-regulatory element-binding protein-1C; n SREBP-1c, nuclear SREBP-1c; FASN, Fatty acid synthase; SCD-1, Stearoyl-CoA desaturase; *Bt, Bacteroides thetaiotaomicron.*

**Figure 7.**
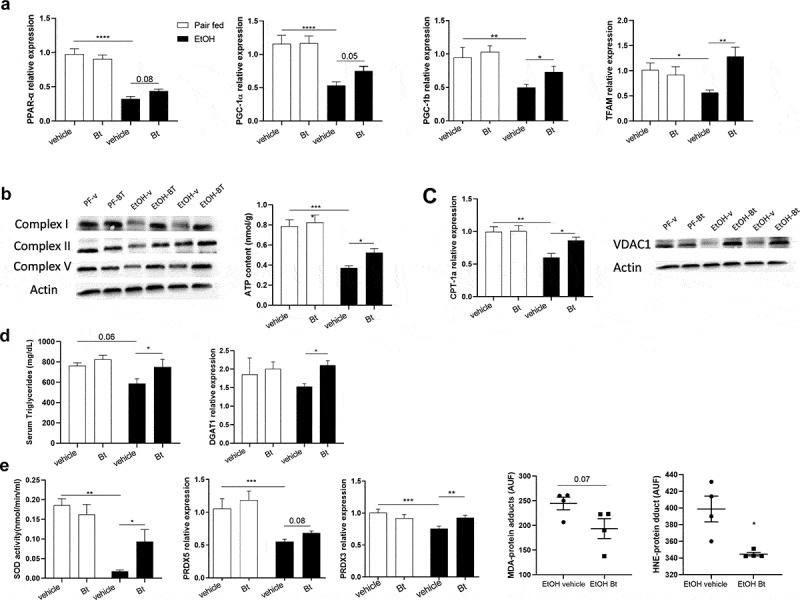
*Bt* improved hepatic mitochondrial fitness and function (a) Liver expression of PPAR-α, TFAM, PGC-1α, PGC-1β fold over *Pair fed vehicle group* and determined by qPCR (Pair fed groups = n4-5; EtOH groups = n6-7). (b) Representative pictures of protein levels of actin, respiratory chain complexes I, II and V determined by western blot analysis (Pair fed groups = n3; EtOH groups = n5), and and hepatic ATP content (Pair fed groups = n4; EtOH groups = n5). (c) Liver expression of CPT-1a fold over *Pair fed vehicle group* and determined by qPCR (Pair fed groups = n4; EtOH groups = n5); and representative pictures of protein levels of actin and VDAC1determined by western blot analysis (Pair fed groups = n3; EtOH groups = n5). (d) Serum levels of Triglycerides (Pair fed groups = n5; EtOH groups = n9), and liver expression of DGAT1 fold over *Pair fed vehicle group* and determined by qPCR (Pair fed groups = n4-5; EtOH groups = n9-10). (e) Hepatic SOD enzymatic activity (Pair fed groups = n4; EtOH groups = n4) and expression of PRDX-3 and −5 fold over *Pair fed vehicle group* and determined by qPCR (Pair fed groups = n5-6; EtOH groups = n9); and serum MDA- and HNE- protein adducts (Pair fed groups = n4; EtOH groups = n4). Data are expressed in mean ± SEM; *p < .05, **p < .01; ***p < .001, ****p < .0001 according to one-Way ANOVA followed by post hoc analysis (Bonferroni test).

Abbreviations: PPAR-α, Peroxisome Proliferator Activated Receptorα; PGC-1α/β, peroxisome proliferator-activated receptor-gamma coactivator-1α/β; TFAM, Transcription Factor A, mitochondrial; CPT-1a, Carnitine Palmitoyltransferase 1a; VDAC1, Voltage-dependent anion-selective channel 1; DGAT1, Diacylglycerol O-Acyltransferase 1; SOD, Superoxide dismutase; PRDX-3/5, Peroxiredoxin 3/5; MDA, Malondialdehyde; HNE, 4Hydroxynonenal; *Bt, Bacteroides thetaiotaomicron.*

Considering the results described above, we have hypothesized that *Bt* improved hepatic steatosis via the recovery of intestinal homeostasis. Interestingly, we found that the liver expression of FGFR4 was up-regulated in EtOH-*Bt* mice (Figure 6a), while GLP-1 receptor (GLP1R) was up-regulated in EtOH-vehicle and EtOH-*Bt* mice (supplementary Fig.5). Analyzing the main lipid metabolism regulators, we found that *Bt* significantly counteracted the reduction of AMPK activity, and the increase of SREBP-1c induced by alcohol (Figure 6b). In particular, *Bt* restored levels of AMPK phosphorylation and normalized total amount and nuclear translocation of SREBP-1c (Figure 6b). AMPK has been described as a target of FGFR4 and GLP1R signaling, and is considered a cellular energetic sensor, able to suppress FA synthesis and stimulate FA oxidation (FAO) to preserve ATP storage.^27^ SREBP-1c is a transcriptional factor of genes involved in FA synthesis; accordingly, FASN and SCD-1 expression was reduced in *Bt*-treated mice compared to EtOH vehicle mice (Figure 6c, d). Overall, these findings suggest that alcohol stimulates FA synthesis by inhibiting AMPK and enhancing SREBP-1c probably by affecting FGF-15 and GLP-1 production. *Bt* administration prevents this by restoring the gut-liver axis. Other AMPK targets are PPAR-α, PGC-1α, and probably PGC-1β, whose primary function is to induce mitochondrial biogenesis, OXPHOS and FAO.^28^ Interestingly, we found that ethanol reduced the expression of all major mitochondrial biogenesis markers such as PPAR-α, PGC-1 α, PGC-1β, and TFAM, while *Bt* significantly restored their expression (Figure 7a). Accordingly, *Bt* recovered protein levels of mitochondrial respiratory chain (RC) complexes I, II, and V, and consequently the cellular bioenergetics improved as shown by ATP levels, which were reduced in EtOH-vehicle and normal in EtOH-*Bt* (Figure 7b). The expression of CPT1a and VDAC1 levels, whose coupled activity is essential for transport of acyl-CoA esters and fuel FAO,^29^ confirmed that FAO was inhibited in ethanol-fed mice and stimulated in *Bt* supplemented ones (Figure 7c). Moreover, PGC-1β is also involved in VLDL exportation.^28^ Part of lipid accumulation in ALD is due to lower lipid exportation.^30^ Accordingly, we found that DGAT1, target of PGC1β involved in VLDL trafficking, was upregulated in EtOH-*Bt* mice and serum triglycerides were lower in EtOH-vehicle mice and normal in EtOH-*Bt* (Figure 7d). Moreover, PGC-1β is known to stimulate the antioxidant response and interestingly, we found that *Bt* restored the SOD activity, PRDX3- and −5 expression, reducing serum lipoperoxidation markers, such as MDA- and HNE-protein adducts (Figure 7e). Taken together, the recovery of *Bt* intestinal abundance in ethanol fed mice permits to counteract the hepatic bioenergetic aberrations induced by alcohol, likely by gut-liver axis rewiring.

## Discussion

The alterations of intestinal microbiota have been described as the driving force of ALD development,^13,31,32^ and in some experimental models, oral supplementation with probiotics, bacteria, and some immunomodulatory molecules attenuated liver alcohol-related liver^16,33,34^ injury. Ethanol ingestion provokes leaky gut and bacterial overgrowth through disruption of mucus layer and tight junction, and downregulation of antimicrobial peptides, such as Reg3b and Reg3g lectins.^35,36^ *Bt* is a gram-negative anaerobe and a prominent member of intestinal bacteria both in humans and mice. Several studies demonstrated *Bt* capacity to modulate host functions such as immunity, mucosal barrier, and nutrients metabolism.^18,21^ In this study, we have further demonstrated that *Bt* drastically decreased in chronically alcohol fed mice and its growth in culture medium is strongly inhibited by ethanol supplementation. Therefore, we investigated whether the recovery of *Bt* intestinal abundance in Lieber-DeCarli fed mice could protect the liver from alcohol exposure. The daily oral administration of *Bt* was effective in restoring alcohol-induced depletion. Interestingly, although the two ethanol-exposed groups showed the same quantity of ethanol in the serum, highlighting equal absorption and metabolization, *Bt*-treated mice presented several benefits. In particular, hepatic steatosis and lipid accumulation were significantly reduced. Perhaps, the shortness of Lieber-DeCarli model does not allow us to appreciate the potential effects of *Bt* on inflammation and liver injury, but only its benefits in alcoholic fatty liver disease (AFLD).

Relevant were the effects on intestinal health, which may explain the improvement of hepatic disease. *Bt* partially restored mucus layer in EtOH-fed mice by increasing muc2 production. Wrzosek et al reported that in a gnotobiotic model *Bt* is involved in goblet cell differentiation and induction of mucins expression such as muc2.^37^ Moreover, a recent study showed that in colitis, goblet cell differentiation and muc2 production are dampened by overexpression of cludin1, which in turn induces MMP9 upregulation and ERK phosphorylation with consequent activation of Notch signaling.^25^ Interestingly, *Bt* supplementation reduced the expression of cldn1 and MMP9, ERK phosphorylation and Hes1 expression (marker of Notch signaling activation), suggesting a potential mechanism by which intestinal bacteria promote mucin production and limit inflammation. Preventing mucus disruption and enhancing antimicrobial peptides expression, *Bt* significantly reduced LPS translocation. Beyond the barrier function, *Bt* showed impact on enterokines production, as it induced a significant increase of GLP1 and restored the ethanol-induced FGF15 reduction. Some studies highlighted the importance of these two hormones (i.e. GLP1, FGF15) in gut liver axis and how bacterial metabolites can modulate their expression promoting or reducing hepatic steatosis in NAFLD.^38^ According to our observation Xie G et al described that chronic alcohol assumption decreased FGF15 intestinal production in rats^39^ and recently, the FXR/FGF15 axis was proposed as a potential treatment in ALD.^40^ It is known that alcohol exposure increases intestinal bile acids absorption and hepatic bile acid production with consequent overload in the gut, liver and serum.^39,41^ In the intestines, bile acids activate FXR which in turn promotes transcription of FGF15/19, which are secreted in the portal circulation. FGF15/19 bind FGFR4 in the liver, suppressing CYP7A1 and bile acid synthesis.^31,42^ Here we found that the expression of FXR targets, IBABP, and in more extent Ost-α and Ost-β, decreased in alcohol-exposed mice and was restored in *Bt*-treated mice, highlighting that *Bt* can prevent the intestinal FXR inhibition occurring in ethanol fed mice. Moreover, the CYP7A1 downregulation in EtOH-*Bt* mice confirmed that the modulation of bile acid metabolism might represent one of the protective mechanisms of *Bt*. GLP-1 is an incretin secreted after a meal ingestion by intestinal L-cells, which are primarily located in the distal ileum and colon. Human and animal studies associated treatments with GLP1 receptor agonists to amelioration of hepatic fat accumulation, insulin resistance and increase of FAs β-oxidation in NAFLD,^43–47^ while in ALD only an effect to reduce ethanol intake was demonstrated both in rodents and humans.^48^ GLP-1 colonic expression can be modulated by bile acids (i.e. deoxycholic and lithocholic acid) via TGR5, or by bacterial derived short chain fatty acids (SCFAs) via GPR43.^38,49^ In our experiments we found that ethanol reduced expression of both TGR5 and GPR43, which were restored by *Bt* supplementation. Therefore, the GLP-1 production in *Bt*-treated mice was likely promoted either by SCFAs and bile acids profile change. Interestingly, analyzing the hepatic metabolic profile we showed that *Bt* administration efficiently prevented the ethanol-induced metabolic abnormality. In particular, *Bt* recovered the ethanol-induced AMPK inhibition, with the consequent normalization of SREBP-1c levels, a known transcriptional factor of FA synthesis genes such as FASN and SCD-1, which accordingly were downregulated in *Bt*-treated mice. Moreover, *Bt* contrasted the ethanol-induced downregulation of mitochondrial biogenesis markers such as PPAR-α, PGC-1α, PGC-1β and TFAM. It is known that the simultaneous activation of these factors stimulates OXPHOS and FAO.^28^ In accordance, we found that *Bt* prevented the RC complexes I, II and V depletion and stimulated FAO. Moreover, the higher antioxidant activity in *Bt*-treated mice might represent an effect of PGC-1 β activation. Interestingly, the entire liver bioenergetic profile could be due to the effect of gut-liver axis perturbations induced by alcohol exposure, and *Bt* improved hepatic metabolism by rewiring intestinal homeostasis. In fact, it is demonstrated that the FGF15/FGFR4 signaling induces AMPK activity, reducing SREBP1c and FA synthesis gene expression.^50^ PPARα can also be activated by FGFR4 activity.^51^ On the other hand, AMPK and PPAR-α can be activated by the GLP1R signaling as well, while FASN and SCD-1 are typically downregulated.^43^ Notably, PGC1β liver expression seems to be modulated by fasting, nutrients, and other unknown gut-derived molecules,^28,52^ underling once more the importance of gut-liver axis and the impact of *Bt* on it. However, it has also been supposed that AMPK activity could enable PGC-1β expression.^28^

In conclusion, ALD development depends on multiple factors and probably the gut-liver axis dysregulation, in terms of bile acid metabolism, enterokine production, and LPS translocation, alters the hepatic capability to regulate the cellular bioenergetics. This results in lipid metabolism abnormality, mitochondrial dysfunction, and oxidative stress. Therefore, it would be conceivable to hypothesize that, as for NASH, a multiple target therapeutic strategy should be tested in ALD, taking into account the possibility of intervening simultaneously on bile acid system, FGF15 and GLP-1 signaling, intestinal barrier, liver mitochondria, and others. The limit of our study is the lack of “loss of function” studies; hence, further investigations will have to clarify the impact of any mechanism involved in the microbiota-intestine-liver axis. However, we have demonstrated that by modulating ethanol-induced microbiota alterations by restoring the intestinal abundance of *Bt*, it is possible to obtain pleiotropic effects by simulating a multiple target therapy. This candidates *Bt* as a potent novel probiotic to treat patients with ALD.

## Materials and methods

### Mouse experiments

An experimental model of ALD was used to study the impact of ethanol introit on intestinal *Bt* and whether oral supplementation with *Bt* could exert beneficial effects. All experiments were performed according to ethical principles and legal laws. 8- to 10-week-old female wild-type (C57BL/6) mice were fed with a Lieber-DeCarli diet containing up to 5% alcohol for 15 days (EtOH-fed) or Lieber-DeCarli diet alone (pair-fed), as shown in Figure 1a and previously described.^33,53^

In order to study the potential properties of *Bt* to protect the liver against ethanol introit, the mice were treated every day (from day 1 to day 14) with *Bt* (3 × 10^9^ bacteria/200 μl phosphate-buffered saline (PBS)) or vehicle (PBS) by oral gavage. Mice were weighed every other day, and drinking amounts were monitored daily. At day 15 mice were sacrificed by being anaesthetized with xylazine (5 mg/kg) and ketamine (100 mg/kg), and blood, liver, and intestinal samples were collected.

### *Quantification of* Bt *in mice feces.*

DNA extraction from feces was performed by DNeasy PowerSoil Kit (QIAGEN, Hilden, Germany) according to manufacturer’s protocol. Following this, the amount of *Bt* DNA was quantified by qPCR with SybrGreen (Eurogentec, Köln, Germany) on MXPro3000 Cycler (Agilent Technology, Waldbronn, Germany). The primers for *Bt* DNA detection were based on 16rDNA gene sequences: forward-GGCAGCATTTCAGTTTGCTTG; reverse-GGTACATACAAAATTCCACACGT. The cycle was performed using 30 ng of fecal DNA, primer concentrations at 250 nM and 60°C as annealing temperature. The standard curve was obtained by serial dilutions of bacterial DNA extracted from *Bt* colonies, and the cycle threshold of each sample was compared with the standard. The quantity of *Bt* DNA in the feces was expressed in Log_10_ ng/g.

In order to further demonstrate the presence of living *Bt* in the intestine of mice treated with oral gavage, mice feces were cultured on blood agar plate, in anaerobic condition, and a qPCR was performed from the growing colonies, showing the presence of *Bt* DNA.

### *Cultivation of* Bt

*Bt* (DSM 2079) was purchased by DSMZ (Leibniz Institute DSMZ-German Collection of Microorganisms and Cell Cultures) and cultured on blood agar (Biomerieux, Marcy l’Etoile, France) at 37°C under anaerobic conditions with GENbox and GENbox anaer systems (Biomeriux, Marcy l’Etoile, France).

### Statistical analysis

GraphPad PRISM v8 (La Jolla, California, USA) was used for statistical analysis. Unpaired two-tailed Student’s t-test and one-way analysis of variance followed by post hoc Bonferroni test were used when appropriate. The results are shown as mean ± standard error of mean (SEM). Data were considered statistically significant when p < .05.

## Supplementary Material

Supplemental Material

## Data Availability

The data that support the findings of this study are available in repository 4TU.ResearchData at https://doi.org/10.4121/16825366.v1.

## Abbreviations

ALD, alcohol-related liver disease; AFLD, alcoholic fatty liver disease; *Bt*, Bacteroides thetaiotaomicron; AH, alcoholic hepatitis; ASH, alcoholic steatohepatitis; FAs, fatty acids; FAO, fatty acid oxidation; FASN, fatty acid synthase; SCD-1, stearoyl-CoA desaturase-1; PPAR-α, peroxisome proliferator-activated receptor-α; PAMPs, pathogen-associated molecular patterns; LPS, lipopolysaccharides; EtOH, ethanol-fed; PBS, phosphate-buffered saline; ALT, alanine aminotransferase; FGF15, fibroblast growth factor 15; IBABP, ileal bile acid-binding protein; Ost-α/β, Organic solute transporter α/β; GLP-1, Glucagon-like peptide-1; G protein-coupled bile acid receptor 1; GPR43, G-protein-coupled receptor 43; Peptide YY; AMPK, 5’ AMP-activated protein kinase; SREBP-1c, Sterol-regulatory element-binding protein-1C; PGC-1α/β, peroxisome proliferator-activated receptor-gamma coactivator-1α/β; TFAM, Transcription Factor A, Mitochondrial; CPT-1a, Carnitine Palmitoyltransferase 1a; VDAC1, Voltage-dependent anion-selective channel 1; DGAT1, Diacylglycerol O-Acyltransferase 1; SOD, Superoxide dismutase; PRDX-3/5, Peroxiredoxin 3/5; MDA, Malondialdehyde; HNE, 4Hydroxynonenal; Muc1, mucin1; Muc2, mucin2; Cldn1, claudin1; MMP9, matrix metallopeptidase 9; Hes1, hairy and enhancer of split-1; GLP1R, GLP-1 receptor; FGFR4, FGF receptor 4.
