# Supplementary material for: Recovery of Bacteroides thetaiotaomicron ameliorates hepatic steatosis in experimental alcohol-related liver disease
Source: Gut Microbes. 2022 Jul 3;14(1):2089006. doi: 10.1080/19490976.2022.2089006 (PMC9255095; doi:10.1080/19490976.2022.2089006)
Supplement: Supplemental Material [file KGMI_A_2089006_SM6293.zip › supplementary.docx]

**Recovery of *Bacteroides thetaiotaomicron* ameliorates hepatic steatosis in experimental alcohol-related liver disease**

Moris Sangineto^1,2^, Christoph Grander^1^, Felix Grabherr^1^, Lisa Mayr^1^, Barbara Enrich^1^, Julian Schwärzler^1^, Marcello Dallio^1,3^, Vidyasagar Naik Bukke^2^, Archana Moola^2^, Antonio Moschetta^4^, Timon E. Adolph^1^, Carlo Sabbà^4^, Gaetano Serviddio^2^ & Herbert Tilg^1^

^1^ Department of Internal Medicine I, Gastroenterology, Hepatology, Endocrinology & Metabolism, Medical University Innsbruck, Innsbruck, Austria

^2^ C.U.R.E. (University Center for Liver Disease Research and Treatment), Liver Unit, Department of Medical and Surgical Sciences, University of Foggia, Foggia, Italy

^3^ Department of Precision Medicine, University of Campania “Luigi Vanvitelli”, Naples, Italy

^4^ Department of Interdisciplinary Medicine, University of Bari, Bari, Italy

**Corresponding author:** Herbert Tilg, Department of Internal Medicine I, Gastroenterology, Hepatology, Endocrinology & Metabolism, Medical University Innsbruck, Innsbruck, Austria.

Email: [Herbert.Tilg@i-med.ac.at](mailto:Herbert.Tilg@i-med.ac.at)

**Conflict of interest:** None.

**Supplementary figures:** 6

**Keywords:** alcohol-related liver disease; Bacteroides thetaiotaomicron; microbiota; steatosis; intestinal barrier

**Materials and methods**

*Expression studies*

Tissue homogenization was performed with TRIzol® reagent (Thermo Fisher Scientific, Waltham, MA) to extract RNA. Reverse Transcription System (Thermo Fisher Scientific, Waltham, MA) was used for reverse transcription and consequently SybrGreen (Eurogentec, Seraing, Belgium) and Mx3000 Cycler (Stratagene California, CA) were used to perform qPCR. The gene expression was normalized to mouse β-actin.

*Western blot analysis*

In total, 40 ug of proteins from liver homogenates were loaded in a 10% SDSPAGE and transferred to a nitrocellulose membrane, blocked for 1.5 h using 5% non-fat dry milk in TBS-t and incubated over night at 4 _C with primary Primary antibodies: Complex II subunit SDHB monoclonal antibody (ab14714); Complex I subunit NDUFB6 monoclonal antibody MS108 (Abcam, ab110258); Anti-ATPB antibody (3D5) - Mitochondrial Marker (Complex V beta subunit) (Abcam, ab14730); Anti-beta-actin antibody (Abcam, ab8227); VDAC1 polyclonal (Merck Millipore, Ab10527); AMPK polyclonal (Merck Millipore, 07-350); pAMPK (Merck Millipore, 09-290); anti-ERK 1/2 antibody (calbiochem, 442704); anti-phosphoERK1/2 antibody (cell signaling technology, 9106); anti-SREBP1c antibody (santa cruz, sc-13551). An incubation of the membrane followed for 1.5 h with a goat HRP-conjugated anti-mouse or Goat HRP-conjugate anti-rabbit (Bio-Rad Laboratories Inc., Segrate (MI), Italy). Bands were developed by the ClarityTM Western ECL Blotting Substrate using a ChemiDoc MP system (Bio-Rad Laboratories Inc., Segrate (MI), Italy), and quantified by the Image LabTM Software.

*Triglyceride analysis*

Frozen (-80°C) liver tissue was homogenized in PBS, adjusting the volume to the weight, followed by 30 minutes incubation at 95° C. Sample was centrifugated at 12000g for 10 minutes at room temperature. Following, supernatant was harvested for triglycerides measurement using the appropriate reagent (Roche, Switzerland). Vials, used for the procedure, were previously coated with fatty-free BSA (Sigma, St. Louis, MO).

*In vitro assay for evaluation of BT growth with ethanol*

In order to investigate whether Bacteroides Thetaiotaomicron (BT) growth was affected by alcohol, LYBHI medium (37 g/L of brain-heart infusion (Sigma-Aldrich, St. Louis, MO) and 5 g/L of yeast extract (Conda)) was supplemented with ethanol (1%; 3%; 5%) and inoculated with BT. After 24h the number of bacteria in the medium was measured by CASY cell counter and analyser (OLS).

*Mucus thickness and goblet cell counting.*

Colon tissue was fixed in Carnoy solution (6 parts of ethanol abs., 6 parts of acetic acid glacial, 1 part of chloroform) and followed by PAS staining.

The sections were analysed with Panoramic Viewer (3DHISTECH, Budapest, Hungary) to quantify mucus thickness (at least 60 measurements for each sample). The number of globet cells per colonic crypt and the mean dimeter were calculated analysing at least 12 crypts for each slide.

*Serum GLP-1 and FGF-15 measurement*

Serum levels of GLP-1 and FGF-15 were measured with GLP-1 ELISA Kit (Clinisciences EM1076) and FGF-15 ELISA kit (MyBioSource) by following manufacturer’s instructions and measuring absorbance with microplate reader FilterMax F5 (Molecular Devices).

*ATP content*

The liver ATP content was assessed by bioluminescence (Enliten ATP assay kit – Promega Corporation, Madison, WI, USA) according to the method of Yang([1](#_ENREF_1)).

*SOD activity*

A commercial kit was used to measure superoxide dismutase activity (706002, Cayman Chemical, Ann Arbor, MI, USA) in freshly prepared liver homogenate according to the manufacturer’s protocols.

*Serum MDA- and HNE-protein adducts*

In mice serum 4-hydroxy-2-nonenal (HNE) and malondialdehyde (MDA) fluorescent adducts were measured by spectrofluorimetric analysis as previously reported([2](#_ENREF_2)). Briefly, 100 mg of liver tissue were homogenized in 100 µL of 1.15% KCl buffer and treated with 500 µL of 10% TCA. The solution was washed three times with 6 mL Ethanol/ether (3:1). After centrifugation at 6000 RPM for 5 min, the pellet was dried and resuspended in distilled water. Fluorescent wave emission was at 460 nm, and excitation at 390 nm for MDA-adducts, while 355 nm was used as excitation for HNE-adducts.

*Histology*

Liver and gut tissues were stained with haematoxylin and eosin (H&E) by the Institute of Pathology at the Medical University of Innsbruck.

A pathologist analysed the h&e liver sections to evaluate hepatic steatosis in a blinded fashion, calculating the percentage of cells with lipid drops accumulation. The Myeloperoxidase immunohistochemistry on liver sections was performed as previously described([3](#_ENREF_3)). While, colon sections were used for immunofluorescence staining of Mucin-2 (Muc-2) as performed in a previous work ([3](#_ENREF_3)). A 340 confocal microscope (Zeiss, Oberkochen, Germany) was used to analyse and take pictures of Muc-2 immunofluorescence.

**Supplementary Fig.1 vehicle and *Bt* treated mice assumed same quantity of alcohol.** (A) Serum ethanol concentrations (Pair fed groups=n3; EtOH groups=n4-5). Data are expressed in mean ± SEM; *p<0.05 according to one-Way ANOVA followed by post hoc analysis (Bonferroni test)


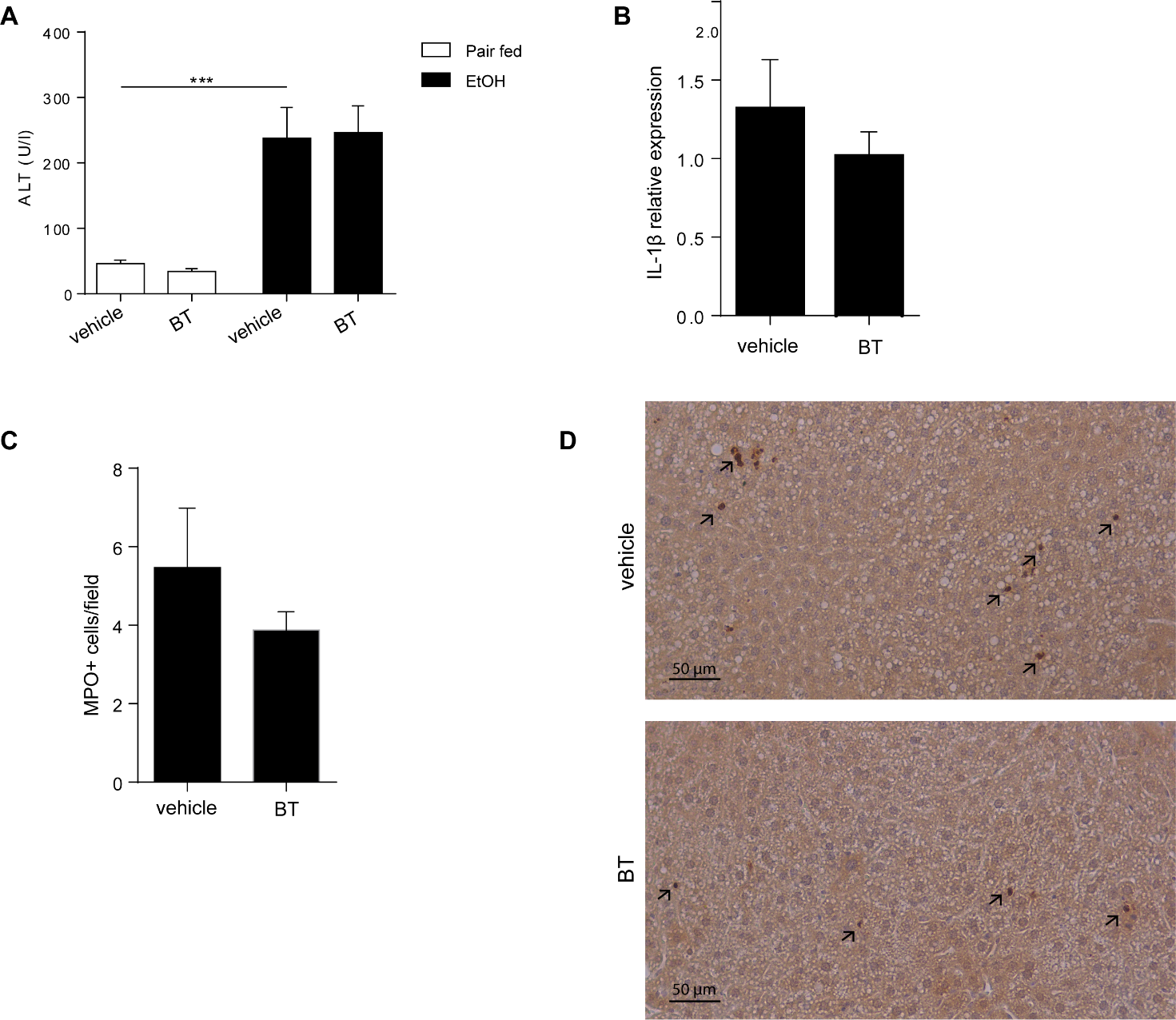


**Supplementary Fig.2 BT only midly reduced hepatic inflammation.** (A) Serum ALT levels (Pair fed groups=n6; EtOH groups=n10). (B) Hepatic expression of IL-1β in EtOH fed mice (n=9-10 per group). (C, D) Representative pictures and quantification of neutrophils in liver tissue of EtOH fed mice, determined by immunoreactivity to MPO (brown; n=5 per group). Data are expressed in mean ± SEM; *p<0.05; **p<0.01; ***p<0.001, according to one-Way ANOVA followed by post hoc analysis (Bonferroni test) or two-tails student’s t-test. BT, *Bacteroides Thetaiotaomicron*; MPO, myeloperoxidase.


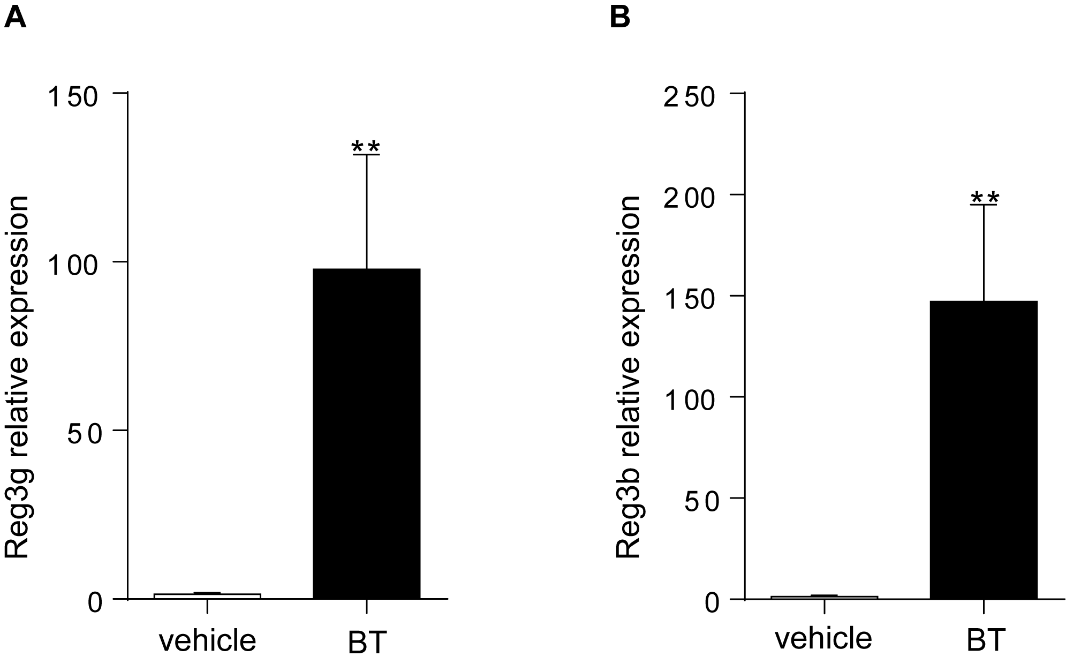


**Supplementary Fig.3 BT up-regulates expression of antimicrobial peptides.** (A, B) Intestinal expression of Reg3g and Reg3b in EtOH fed mice and determined by qPCR (n=8-10 per group). Data are expressed in mean ± SEM; *p<0.05; according to two-tails student’s t-test. Reg3 -g -b, Regenerating islet-derived protein 3 -g -b; BT, *Bacteroides Thetaiotaomicron*.

**Supplementary Fig.4 Bt normalized hepatic CYP7A1 expression.** Liver expression of CYP7A1fold over Pair fed groups and determined by qPCR (n=4 per group). Data are expressed in mean ± SEM; *p<0.05 according to one-Way ANOVA followed by post hoc analysis (Bonferroni test). Bt, *Bacteroides Thetaiotaomicron*; CYP7A1, Cytochrome P450 Family 7 Subfamily A Member 1.

**Supplementary Fig.5 GLP1R expression increased in EtOH-fed mice.** Liver expression of GLP1R fold over Pair fed groups and determined by qPCR (n=6-9 per group). Data are expressed in mean ± SEM; ****p<0.0001according to one-Way ANOVA followed by post hoc analysis (Bonferroni test). Bt, *Bacteroides Thetaiotaomicron*; GLP1R, Glucagon loke peptide 1 receptor.


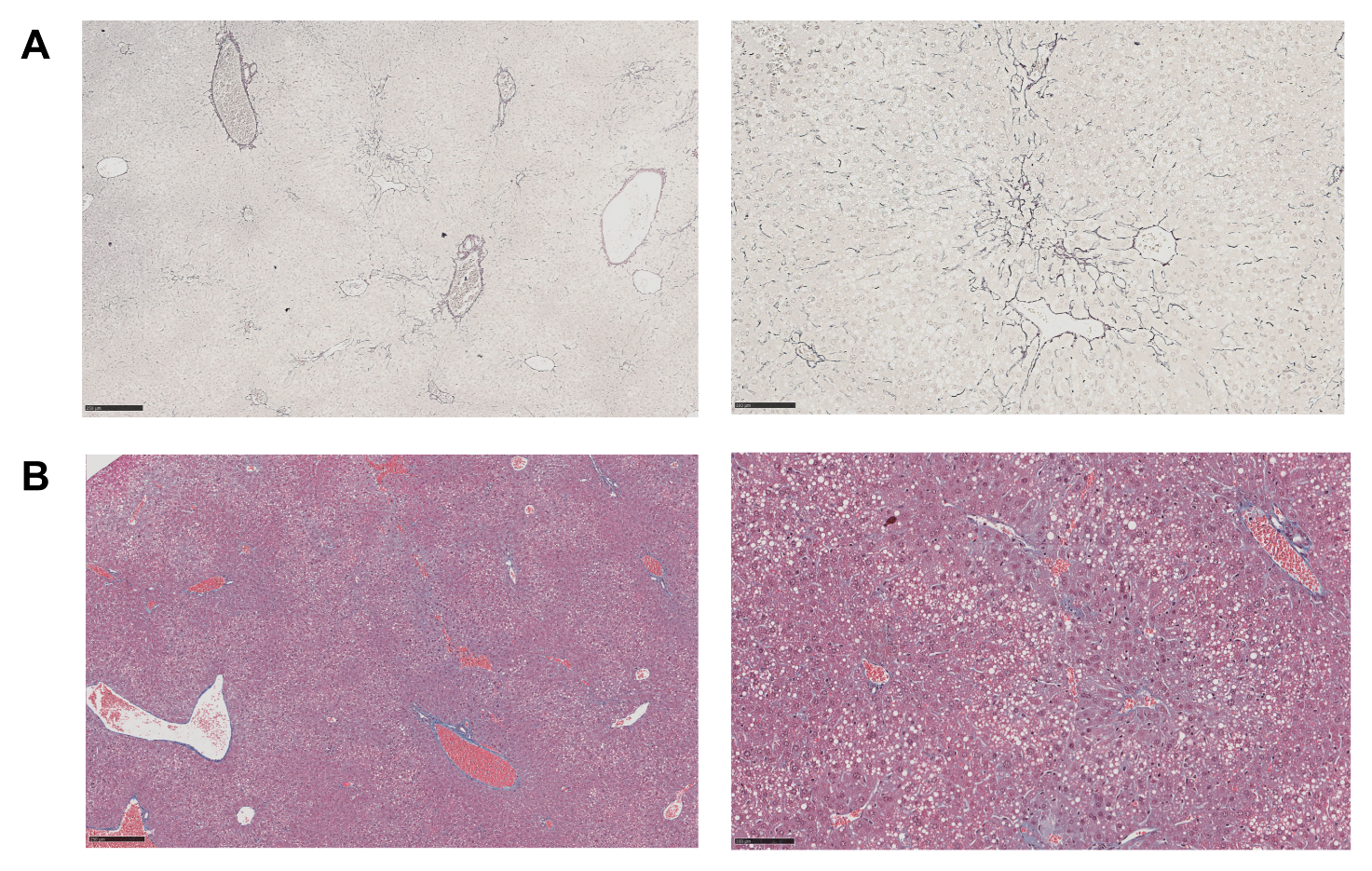


**Supplementary Fig.6 Mild fibrosis in ethanol-fed mice.** (A) Liver sections coloured with Gomori stain. (B) Liver sections stained with Masson’s trichrome. The staining indicates that in this alcohol-related liver disease model, only a very mild fibrosis develops.

Reference List

1. Yang NC, Ho WM, Chen YH, Hu ML. A convenient one-step extraction of cellular ATP using boiling water for the luciferin-luciferase assay of ATP. Anal Biochem. 2002;306(2):323-7.

2. Sangineto M, Bukke VN, Bellanti F, Tamborra R, Moola A, Duda L, et al. A Novel Nutraceuticals Mixture Improves Liver Steatosis by Preventing Oxidative Stress and Mitochondrial Dysfunction in a NAFLD Model. Nutrients. 2021;13(2).

3. Grander C, Adolph TE, Wieser V, Lowe P, Wrzosek L, Gyongyosi B, et al. Recovery of ethanol-induced Akkermansia muciniphila depletion ameliorates alcoholic liver disease. Gut. 2018;67(5):891-901.
