# Supplementary material for: Recovery of Bacteroides thetaiotaomicron ameliorates hepatic steatosis in experimental alcohol-related liver disease
Source: Gut Microbes. 2022 Jul 3;14(1):2089006. doi: 10.1080/19490976.2022.2089006 (PMC9255095; doi:10.1080/19490976.2022.2089006)
Supplement: Supplemental Material [file KGMI_A_2089006_SM6293.zip › supplementary_figure_captions.docx]

**Supplementary Fig.1 vehicle and *Bt* treated mice assumed same quantity of alcohol.** (A) Serum ethanol concentrations (Pair fed groups=n3; EtOH groups=n4-5). Data are expressed in mean ± SEM; *p<0.05 according to one-Way ANOVA followed by post hoc analysis (Bonferroni test).

**Supplementary Fig.2 BT only midly reduced hepatic inflammation.** (A) Serum ALT levels (Pair fed groups=n6; EtOH groups=n10). (B) Hepatic expression of IL-1β in EtOH fed mice (n=9-10 per group). (C, D) Representative pictures and quantification of neutrophils in liver tissue of EtOH fed mice, determined by immunoreactivity to MPO (brown; n=5 per group). Data are expressed in mean ± SEM; *p<0.05; **p<0.01; ***p<0.001, according to one-Way ANOVA followed by post hoc analysis (Bonferroni test) or two-tails student’s t-test. BT, *Bacteroides Thetaiotaomicron*; MPO, myeloperoxidase.

**Supplementary Fig.3 BT up-regulates expression of antimicrobial peptides.** (A, B) Intestinal expression of Reg3g and Reg3b in EtOH fed mice and determined by qPCR (n=8-10 per group). Data are expressed in mean ± SEM; *p<0.05; according to two-tails student’s t-test. Reg3 -g -b, Regenerating islet-derived protein 3 -g -b; BT, *Bacteroides Thetaiotaomicron*.

**Supplementary Fig.4 Bt normalized hepatic CYP7A1 expression.** Liver expression of CYP7A1fold over Pair fed groups and determined by qPCR (n=4 per group). Data are expressed in mean ± SEM; *p<0.05 according to one-Way ANOVA followed by post hoc analysis (Bonferroni test). Bt, *Bacteroides Thetaiotaomicron*; CYP7A1, Cytochrome P450 Family 7 Subfamily A Member 1.

**Supplementary Fig.5 GLP1R expression increased in EtOH-fed mice.** Liver expression of GLP1R fold over Pair fed groups and determined by qPCR (n=6-9 per group). Data are expressed in mean ± SEM; ****p<0.0001according to one-Way ANOVA followed by post hoc analysis (Bonferroni test). Bt, *Bacteroides Thetaiotaomicron*; GLP1R, Glucagon loke peptide 1 receptor.

**Supplementary Fig.6 Mild fibrosis in ethanol-fed mice.** (A) Liver sections coloured with Gomori stain. (B) Liver sections stained with Masson’s trichrome. The staining indicates that in this alcohol-related liver disease model, only a very mild fibrosis develops.
