# Supplementary figures and images for: Recovery of Bacteroides thetaiotaomicron ameliorates hepatic steatosis in experimental alcohol-related liver disease
Source: Gut Microbes. 2022 Jul 3;14(1):2089006. doi: 10.1080/19490976.2022.2089006 (PMC9255095; doi:10.1080/19490976.2022.2089006)

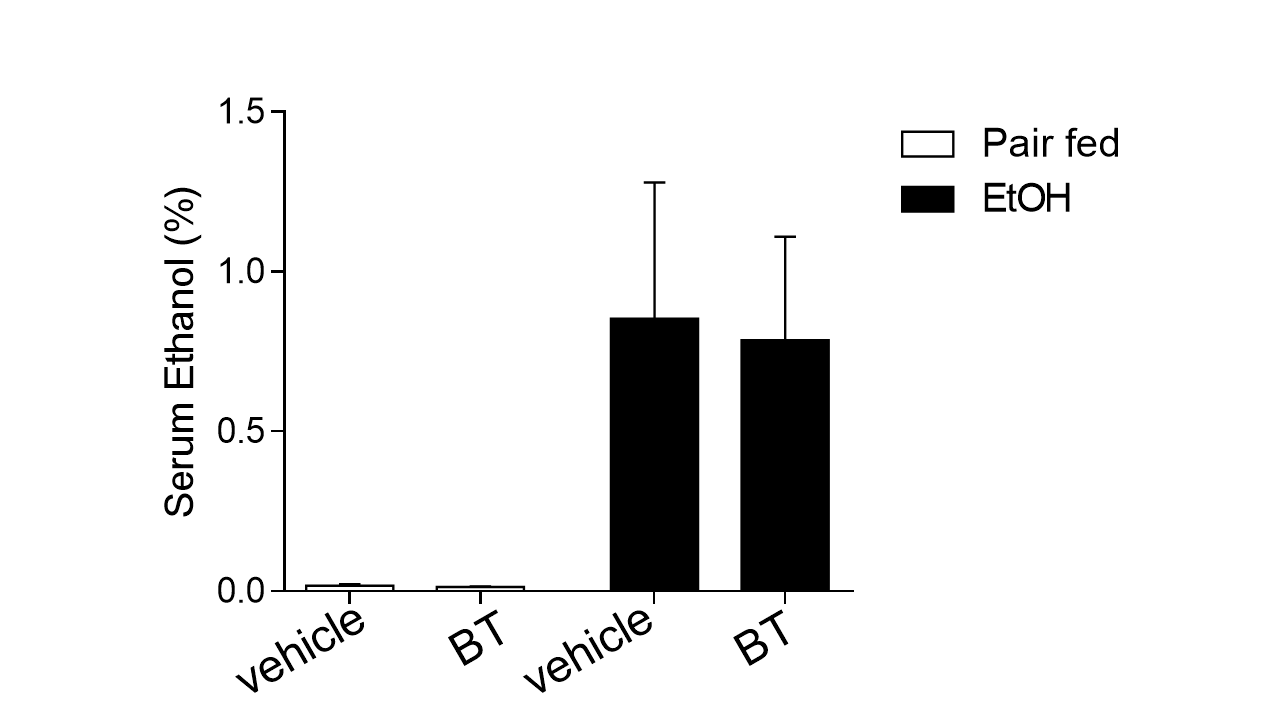

Supplement: Supplemental Material [file KGMI_A_2089006_SM6293.zip › Supplementary_figure1.tif]

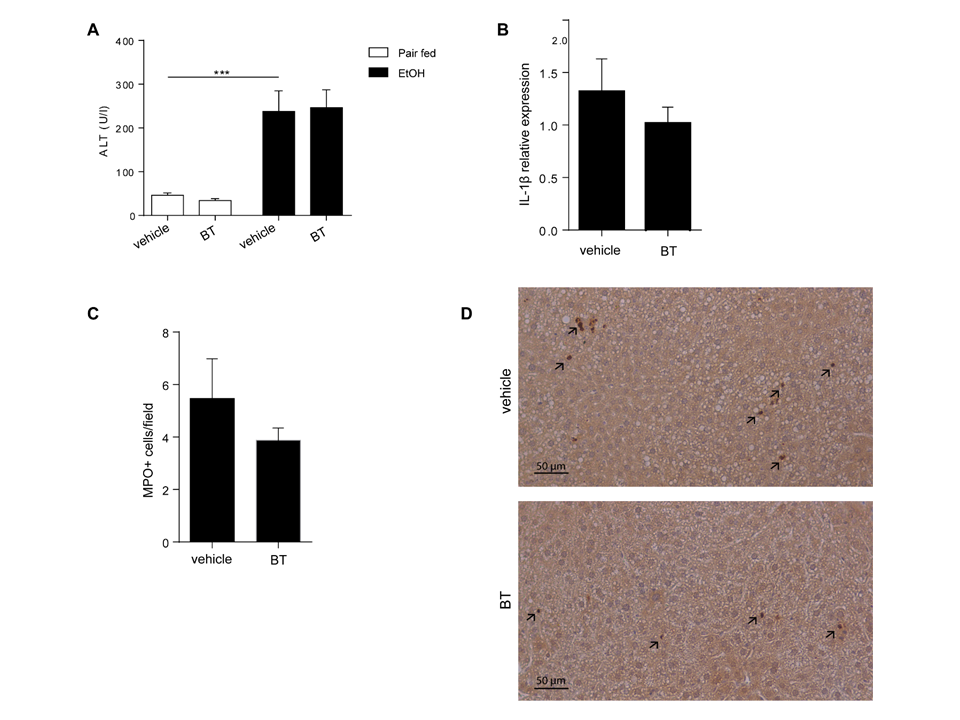

Supplement: Supplemental Material [file KGMI_A_2089006_SM6293.zip › Supplementary_figure_2.tif]

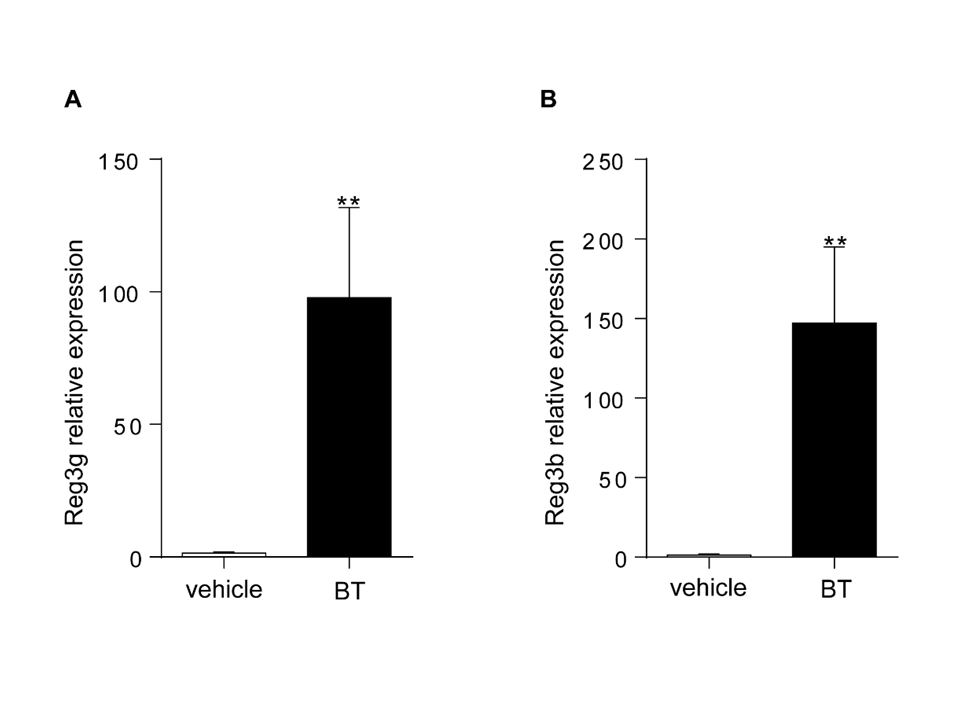

Supplement: Supplemental Material [file KGMI_A_2089006_SM6293.zip › Supplementary_figure_3.tif]

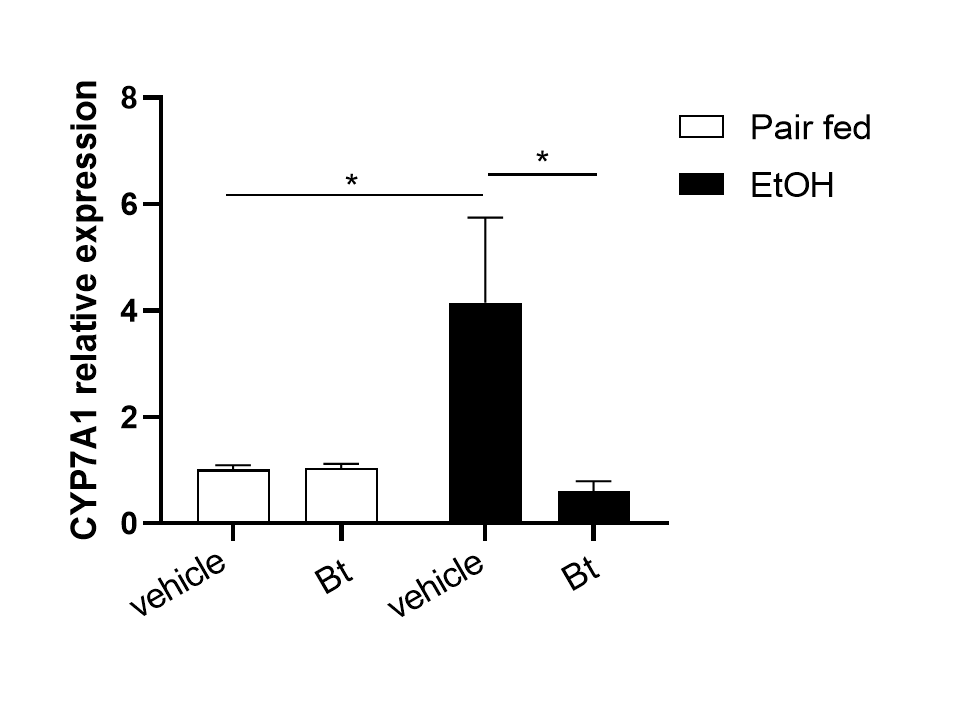

Supplement: Supplemental Material [file KGMI_A_2089006_SM6293.zip › Supplementary_figure_4.tif]

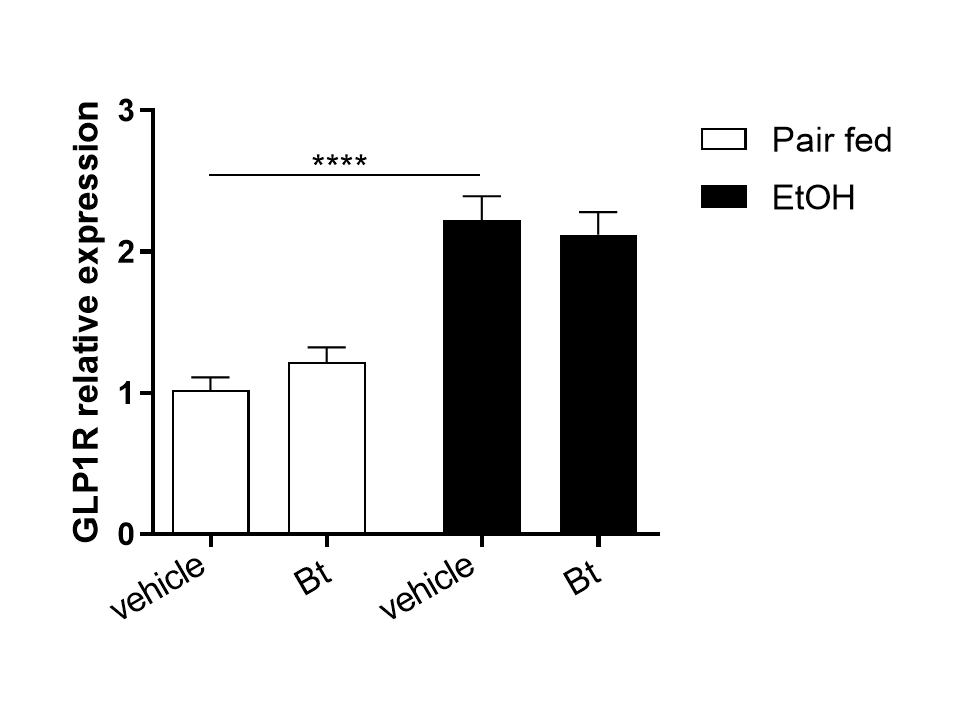

Supplement: Supplemental Material [file KGMI_A_2089006_SM6293.zip › Supplementary_figure_5.tif]

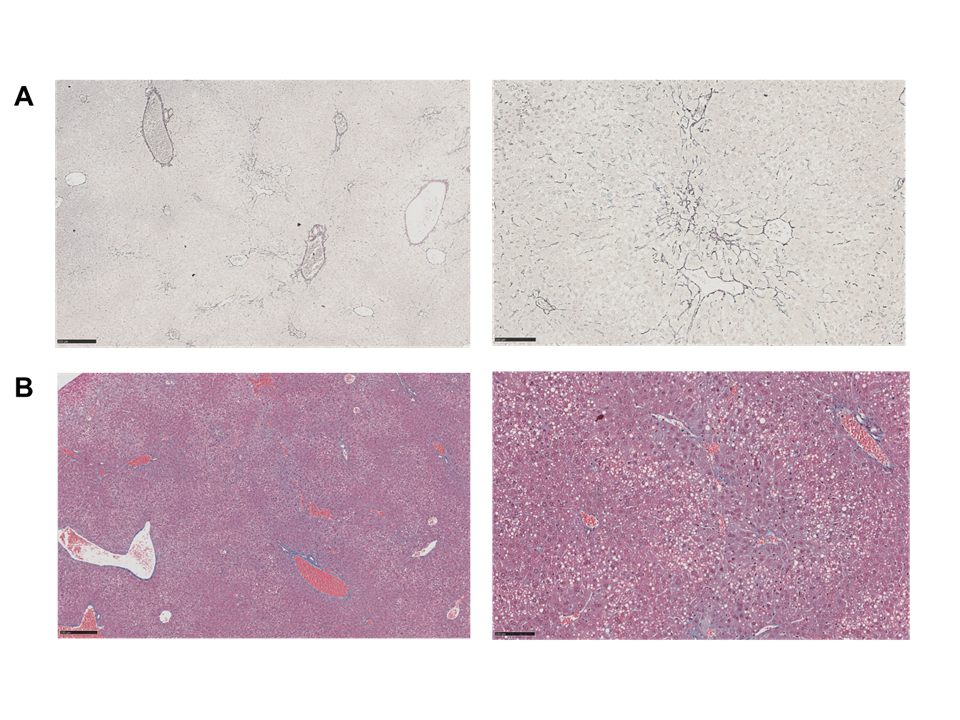

Supplement: Supplemental Material [file KGMI_A_2089006_SM6293.zip › Supplementary_figure_6.tif]
